# The direct effect of type 2 diabetes on neurodegenerative processes in the absence of other cardiovascular risk factors

**DOI:** 10.1002/alz.089136

**Published:** 2025-01-09

**Authors:** Joseph Nowell, Steve Gentleman, Paul Edison

**Affiliations:** ^1^ Imperial College London, London UK

## Abstract

**Background:**

Diabetes is associated with an increased risk of developing neurodegenerative conditions, including Alzheimer’s disease. Abnormal insulin signalling can lead to impaired phosphorylation of protein kinase B and activation of phosphatidylinositol 3‐kinase, resulting in hyperphosphorylation of tau and activation of inflammatory pathways. However, people living with diabetes commonly exhibit comorbid cardiovascular risk, which are also linked with cognitive decline. Here we sought to establish the influence of diabetes on global brain atrophy in the absence of comorbid cardiovascular disease.

**Method:**

Participants diagnosed with diabetes and a matched sample of non‐diabetic control participants were enrolled from the UK biobank database. To reduce the influence of confounding risk factors, diabetic and control participants with additional cardiovascular risk were excluded. Participants diagnosed with cardiovascular/heart problems including high blood pressure, cardiac arrest, stroke, and angina were excluded. Non‐diabetic controls were matched based on age, sex and APOE genotype to enhance statistical precision. Voxel‐based morphometry was performed on T1‐weighted structural magnetic resonance images to evaluate the macrostructural changes associated with diabetes across the cortex. A voxel‐wise t‐test was performed between diabetic and non‐diabetic controls implemented on FSL.

**Result:**

A total of 503 diabetic participants and a matched sample of 503 control participants met the inclusion criteria and were enrolled on the study. Even in the absence of comorbid cardiovascular disease, participants with diabetes exhibited widespread reductions in grey matter volume in comparison to non‐diabetic participants. Volumetric reductions were demonstrated in 97% of grey matter (volume = 191605 mm³), indicating neurodegeneration in all major brain lobes. Reductions of brain volume in diabetic participants remained following family‐wise error correction.

**Conclusion:**

In the absence of other comorbid cardiovascular diseases, type 2 diabetes mellitus induces widespread neurodegeneration in cortical regions. Impairment to insulin signalling and the initiation of the inflammation response may initiate neurodegenerative processes resulting in the loss of brain cells. The widespread nature of atrophy indicates the potential risk for future cognitive impairment. Targeting molecular mechanisms involved in diabetes, including insulin resistance and inflammation, are important to prevent the development of neurodegenerative conditions. This exemplifies the potential for antidiabetic agents for the treatment of neurodegenerative diseases.

